# The Signaling Pathways Controlling the Efficacy of Glioblastoma Therapy

**DOI:** 10.32607/actanaturae.11623

**Published:** 2022

**Authors:** N. S. Vasileva, A. B. Ageenko, V. A. Richter, E. V. Kuligina

**Affiliations:** Institute of Chemical Biology and Fundamental Medicine SB RAS, Novosibirsk, 630090 Russia

**Keywords:** glioblastoma, oncolytic viruses, VV-GMCSF-Lact, cancer stem cells, mechanisms of glioblastoma resistance

## Abstract

The resistance of glioblastoma to existing therapies puts limits on quality-of-life improvements and patient survival with a glioblastoma diagnosis. The development of new effective glioblastoma therapies is based on knowledge about the mechanisms governing tumor resistance to therapeutic agents. Virotherapy is one of the most actively developing approaches to the treatment of malignant neoplasms: glioblastoma in particular. Previously, we demonstrated that the recombinant vaccinia virus VV-GMCSF-Lact exhibits *in vitro* cytotoxic activity and *in vivo *antitumor efficacy against human glioblastoma. However, the studied glioblastoma cell cultures had different sensitivities to the oncotoxic effect of the virus. In this study, we investigated cancer stem cell (CSC) surface markers in glioblastoma cells with different sensitivities to VV-GMCSFLact using flow cytometry and we assessed the levels of proteins affecting viral entry into cells and virus infection efficiency by western blotting. We showed that cell cultures more sensitive to VV-GMCSF-Lact are characterized by a greater number of cells with CSC markers and a lower level of activated Akt kinase. Akt probably inhibits lactaptin-induced apoptosis in virus-resistant cells. Hence, we suggest that the sensitivity of glioblastoma cells to the oncotoxic effect of VV-GMCSF-Lact is determined by the nature and extent of the disturbances in cell death regulation in various cultures. Further investigation of the factors affecting glioblastoma resistance to virotherapy will test this hypothesis and identify targets for antitumor therapy, combined with VV-GMCSF-Lact.

## INTRODUCTION

Glioblastoma is the most malignant tumor of the central nervous system, which is characterized by a low patient survival rate. Standard glioblastoma therapy, which involves maximal surgical resection followed by radiation therapy and/or chemotherapy, neither improves the quality of life nor increases the survival rate of patients with this diagnosis.

The key challenge to effective glioblastoma treatment is tumor resistance to existing therapies. Today, the scientific and medical community is developing and promoting various approaches to glioblastoma therapy that are based on the inhibition of target molecules, immunotherapy, and other methods. Therapy with oncolytic viruses, an approach to the immunotherapy of tumors, and gliomas in particular, is being actively developed [1].

Previously, a team of researchers from the Institute of Chemical Biology and Fundamental Medicine SB RAS and State Research Center of Virology and Biotechnology VECTOR designed a recombinant vaccinia virus strain, VV-GMCSF-Lact. VV-GMCSF-Lact contains deletions of the thymidine kinase and growth factor genes in whose regions the GM-CSF and oncotoxic protein lactaptin genes are inserted. Deletion of these genes reduces the virus virulence for healthy cells and significantly increases its selectivity for tumor cells. Expression of GM-CSF promotes an antitumor immune response, whereas expression of lactaptin, a fragment of human milk kappa-casein, induces the apoptotic death of tumor cells. VV-GMCSFLact was previously shown to exhibit high cytotoxic activity against human tumor cells of different histogenesis* in vitro *and significant antitumor activity against human breast cancer and glioblastoma *in vivo* [2, 3].

However, different glioblastoma cell cultures, both immortalized and derived from patient tumor samples (patient-derived cell cultures), have different sensitivities to VV-GMCSF-Lact. Glioblastoma is known to be characterized by intertumor and intratumor heterogeneity. Some molecular glioblastoma subtypes, such as proneural, are more sensitive to radiotherapy and temozolomide chemotherapy; however, certain glioblastoma subtypes, and the mesenchymal molecular subtype in particular, are resistant to standard therapy [4, 5]. In this case, the issue of tumor cell resistance to viral therapy remains open [6, 7].

The vaccinia virus, which was used to design VVGMCSF- Lact, is able to penetrate target cells via fusion with the cell membrane or (at low pHs) endosomal membranes [8, 9]. Furthermore, phosphatidylserine present on the membrane of viral particles was shown to facilitate virus entry into the cell via macropinocytosis by mimicry of apoptotic bodies [10]. These entry pathways require rearrangement of the target cell cytoskeleton; therefore, their efficiency may depend on the status of various cellular signaling pathways: e.g., the PI3K/Akt pathway. In addition, all macropinocytosis stages require p21-activated kinase (PAK1) that is involved in cytoskeleton reorganization and microtubule dynamics [11].

In this study, we investigated the factors that control the cytotoxic effect of VV-GMCSF-Lact against glioblastoma cells with different sensitivities to the virus. We assessed the abundance of glioblastoma cancer stem cell (CSC) markers in immortalized U87 MG and U343 MG cell lines and patient-derived BR1.20 and BR3.20 cell cultures and measured the levels of proteins affecting the viral infection efficiency in tumor cells – elements of the PI3K/Akt signaling pathway, and PAK1. Glioblastoma cell cultures sensitive to the oncotoxic effect of VV-GMCSF-Lact were shown to be characterized by a greater number of cells carrying CSC markers and a lower (compared to resistant cells) level of activated Akt protein kinase capable of inhibiting lactaptin-induced apoptosis.

## EXPERIMENTAL

**Glioblastoma cell culture**

The human glioblastoma cell cultures U87 MG, U343 MG, BR1.20, and BR3.20 were taken from the cell culture collection of the Institute of Chemical Biology and Fundamental Medicine SB RAS (Novosibirsk, Russia).

Cells of immortalized U87 MG and U343 MG cultures were cultured in an alpha-MEM medium supplemented with 10% FBS, 2 mM *L*-glutamine, and an antibiotic/antimycotic solution (100 U/mL penicillin, 100 mg/mL streptomycin sulfate, and 0.25 µg/mL amphotericin).

Cells of patient-derived BR1.20 and BR3.20 cultures were cultured in a DMEM/F12 medium supplemented with 10% FBS, 4 mM *L*-glutamine, and an antibiotic/ antimycotic solution (200 U/mL penicillin, 200 mg/mL streptomycin sulfate, and 0.5 µg/mL amphotericin).

All the cell cultures were maintained in a CO_2_ incubator at 37.0 ± 1.0°C in an atmosphere of 5.0 ± 0.5% CO_2_.

**Flow cytometry**

The cells that had reached 60–80% confluence were harvested from the culture dish and incubated with anti-human CD15 monoclonal antibodies conjugated to Alexa Fluor 647 (R&D Systems, USA) and anti-human CD171 monoclonal antibodies conjugated to PE (R&D Systems), according to the manufacturer’s protocol. Analysis was performed using a FACSCanto II flow cytometer (BD Biosciences, USA). Data were analyzed using the FACSDiva software (BD Biosciences).

**Western blot analysis**

The levels of the p85α, p110α, pAk^tSer473^, pAkt^Thr308^, and pPAK^1Ser199/204^ proteins, before and after exposure to the virus (after 0.5, 1, 2, 6, and 12 h), were assessed using the Western blot analysis. The multiplicity of infection was 1 PFU/cell.

The cells were incubated with the virus. Then, cell lysates were produced using a RIPA buffer (1% NP40, 150 mM NaCl, 0.1% SDS, 50 mM Tris-HCl, pH 7.4) in the presence of protease and phosphatase inhibitors (Pierce Phosphatase Inhibitor Mini Tablets, Thermo Scientific, USA) and a cOmplete™ Protease Inhibitor Cocktail (Sigma-Aldrich, USA). The protein concentration in the resulting lysates was measured using a commercial Modified Lowry Protein Assay Kit (Thermo Scientific), according to the manufacturer’s protocol. The proteins were separated by denaturing 10% polyacrylamide gel electrophoresis using a vertical electrophoresis chamber. “Wet” transfer of the proteins from a gel onto a nitrocellulose membrane (0.45 µm) was performed in a NuPAGE Transfer Buffer (Invitrogen, USA) at a direct current of 400 mA for 1 h. Membranes were treated with antibodies using an IBind Western Device (Bio-Rad, USA). The proteins were detected using a Novex® ECL Chemiluminescent Substrate Reagent Kit (Invitrogen) and an Amersham™ Imager 600 System. For normalization, the membranes were stained with anti-β-actin recombinant rabbit monoclonal antibodies.

## RESULTS AND DISCUSSION

**CD15- and CD171-positive cells in U87 MG, U343 MG, BR1.20, and BR3.20 human glioblastoma cultures**

Previously, we demonstrated that the recombinant vaccinia virus VV-GMCSF-Lact with deletions of the viral thymidine kinase and growth factor genes in whose regions the human GM-CSF and oncotoxic protein lactaptin gene are inserted exhibited high cytotoxic activity and antitumor efficacy against both immortalized and patient-derived cell cultures. In that case, the studied cells had different sensitivities to the virus [3].

**Table:** Cytotoxic activity of VV-GMCSF-Lact against glioma cells

Cell culture	IC_50_^*^, PFU/cell
U87 MG	0.1
U343 MG	0.06
BR1.20	0.006
BR3.20	0.02

^*^IC_50_ is the virus concentration causing 50% cell death.

In this study, we investigated some factors that can influence the effect of the virus on tumor cells. U87 MG and U343 MG immortalized glioblastoma cell lines and BR1.20 and BR3.20 cell cultures derived from patient tumor samples (patient-derived cell cultures) were used for this purpose. The analyzed cells exhibit different sensitivities to the oncolytic virus VVGMCSF- Lact (*Table*).

**Fig. 1:**
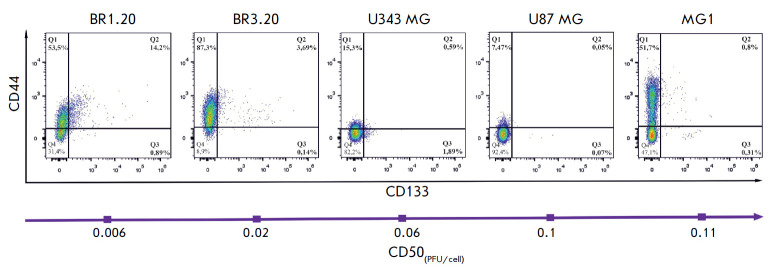
The expression profiles of CD133 and CD44 and their co-expression in MG1, BR1.20, BR3.20, U343 MG, and U87 MG cell cultures. Cell suspensions were incubated with PE-conjugated anti-CD133 and APC-conjugated anti-CD44 antibodies and analyzed by flow cytometry. The CD44-positive cell population is displayed in the upper quadrants (Q1, Q2); The CD133-positive population is represented in the right quadrants (Q2, Q3). Cells positive for both markers are presented in the upper right quadrant (Q2). The purple arrow indicates a decrease in the sensitivity of the studied cell cultures to VV-GMCSF-Lact [3]

The differences in the cytotoxic effect of the virus on the cells may be associated primarily with the diversity of the origin of the studied cell cultures. Glioblastoma is known to belong to a heterogeneous group of malignancies with different responses to therapy [12]. In addition, these neoplasms are characterized by intratumoral heterogeneity at the molecular level and a complex cellular organization [13]. According to the hierarchical model, glioblastoma CSCs are at the top of the hierarchy and significantly contribute to tumor therapy resistance [14]. Thus, the Notch signaling pathway, which plays an important role in maintaining the CSC phenotype, promotes the development of radiotherapy resistance by these cells via the activation of the PI3K/AKT and Bcl-2 pathways, which are important regulators of cell growth and survival [15]. Previously, we showed that glioblastoma cell cultures more sensitive to VV-GMCSFLact contain more CD133+ and CD133+/CD44+ cells (*Fig. 1*) [3].

**Fig. 2:**
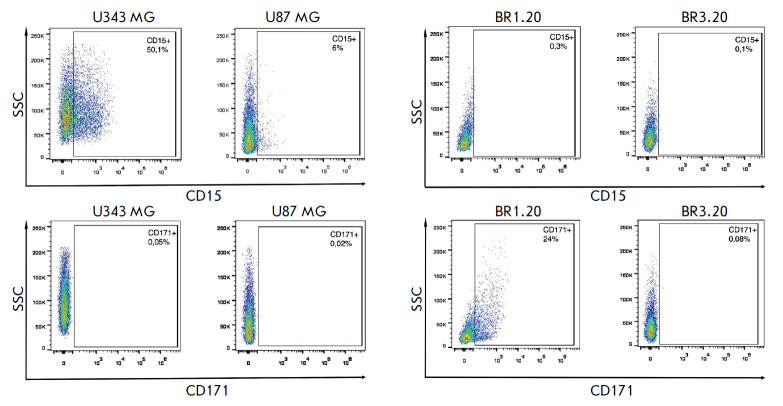
Expression profiles of the CD15 and CD171 markers in U87 MG, U343 MG, BR1.20, and BR3.20 cell cultures. Cell suspensions were incubated with PE-conjugated anti-CD171 and FITC-conjugated anti-CD15 antibodies and analyzed by flow cytometry

Using flow cytometry, we assessed the abundance of other CSC markers, CD15 and CD171 [16, 17], in U87 MG, U343 MG, BR1.20, and BR3.20 cell cultures with different sensitivities to VV-GMCSF-Lact (*Fig. 2*).

CD15, also known as SSEA-1 or Lewis X, is a carbohydrate adhesion molecule usually present on many types of pluripotent stem cells [18]. In the present study, CD15-positive cells were present in U87 MG and U343 MG immortalized cell cultures (6 and 50.1%, respectively). In this case, the number of CD133- positive cells in these cultures was significantly lower, or no such cells were detected [3]. In 2009, M. Son et al. showed that patient-derived glioblastoma cell cultures may not contain CD133-positive cancer stem cells. However, these cultures contained cells that had properties similar to those of stem cells and exhibited a tumorigenic potential when transplanted to immunodeficient mice [19]. Hence, CD15 can be considered an alternative to CD133 in the isolation and characterization of glioblastoma stem cells.

CD171, or L1CAM, belongs to the family of immunoglobulin- like cell adhesion molecules and plays an important role in the development of neural cells, survival, and migration of tumor cells [20, 21]. CD171 mediates the development of radio- and chemoresistance in glioblastoma cells [22, 23]. It should be noted that BR1.20 is the only cell culture in this study containing CD171-positive cells.

Thus, the BR1.20 cell culture was the most sensitive to VV-GMCSF-Lact. Its cells are characterized by a higher content not only of CD133 and CD44, but also CD171 that is involved in maintaining the survival and clonogenicity of CD133-positive CSCs due to positive regulation of Olig2, thus leading to a reduced expression of the tumor suppressor p21 [24]. CD133, known as prominin-1, plays an important role in cell growth, proliferation and the pathophysiology of tumors [25]. CD133+ cells were shown to be radiotherapy resistant due to a more efficient repair system [26]. Phosphorylation of the CD133 cytoplasmic domain results in its binding to p85 (the PI3K regulatory subunit), followed by the activation of the PI3K/Akt signaling pathway [27]. According to the literature, activation of the PI3K/Akt signaling pathway is one of the leading processes controlling the entry of VACV viral particles into the cell and virus replication at the early stages of infection [28, 29, 30].

**p85α, p110α, pAkt^Ser473^, pAkt^Thr308^, and pPAK1^Ser199/204^ protein levels in U87 MG and U343 MG cells before and after exposure to the virus**

The vaccinia virus is able to enter the host cell both via complete membrane fusion and pH-dependent endocytosis and via macropinocytosis through the interaction between phosphatidylserine residues on the viral membrane and the G-protein-coupled receptors of the cell, leading to the activation of downstream signaling pathways, such as PI3K/Akt, reorganization of the host cell cytoskeleton, and subsequent virus entry into the cell. Inhibition of PI3K was shown to decrease the number of virions entering the cell [10]. In addition, all macropinocytosis stages involve P21- activated kinase PAK1, whose transfer to the plasma membrane leads to the activation of many of the effectors necessary for macropinosome formation [11].

**Fig. 3:**
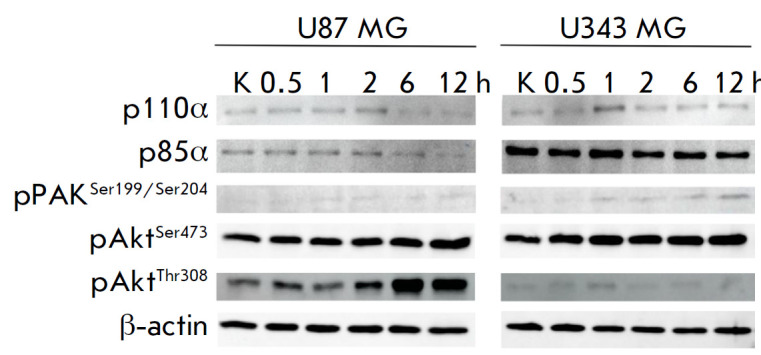
Analysis of the p85α, p110α, pAKT^Ser473^, pAKT^Thr308^, and pPAK1Ser^199/204^ proteins in the cells of immortalized cultures U87 MG and U343 MG. Western blot. Lanes: K – lysates of control cells; 0.5, 1, 2, 6, and 12 h – lysates of cells incubated with VV-GMCSF-Lact for different periods of time

**Fig. 4:**
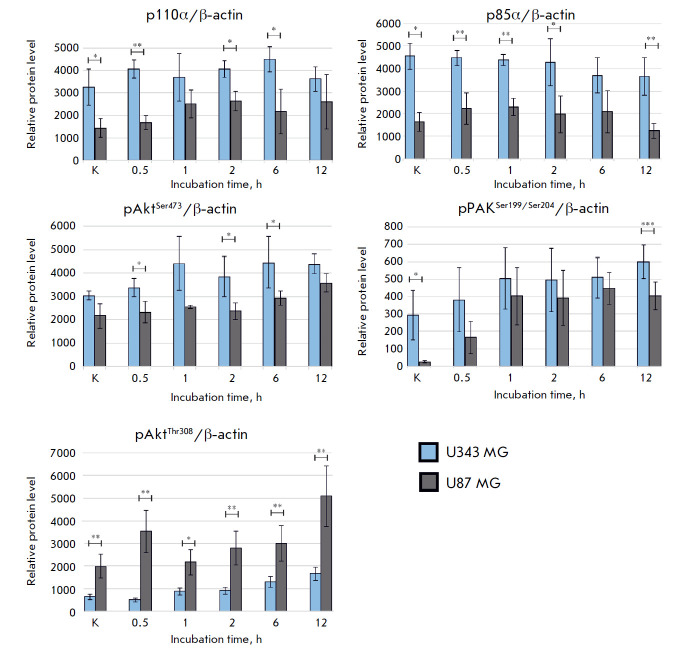
The relative levels of the p85α, p110α, pAKT^Ser473^, pAKT^Thr308^, and pPAK1Ser^199/204^ proteins in the cells of immortalized cultures U87 MG and U343 MG before and after incubation with VV-GMCSFLact (* *p * < 0.05, ** *p* < 0.01, *** *p* < 0.001)

Assessment of the levels of p85α and p110α (the PI3K regulatory and catalytic subunits) in U87 MG and U343 MG cells showed that the relative levels of these proteins both at the point of control and at different virus incubation times are higher in U343 MG cells that are more sensitive to VV-GMCSF-Lact (*Fig. 3, Fig. 4*). Higher p85α and p110α levels are probably associated with the formation of a larger number of PI3K heterodimers and, accordingly, the activation of the PI3K/Akt signaling cascade that, according to published data, is involved not only in vaccinia virus entry into the cell, but also in the early stages of viral replication [28]. In addition, the U343 MG cells most sensitive to the virus have a higher level of pAktSer473 and pPAK1Ser199/204 (PAK1 autophosphorylation at these sites prevents the kinase from being converted to an inactive conformation [31]). At the same time, the levels of these proteins rose with the duration of the incubation of both cell lines with the virus. PAK1 is involved in cytoskeleton reorganization and microtubule dynamics, mediating cell membrane blebbing [32]; inhibition of this enzyme reduces the efficiency of cell infection with the vaccinia virus [10]. Akt is phosphorylated at serine 473 by the mTORC2 complex [33]. The conserved poxvirus protein F17 is known to sequester Raptor and Rictor and disrupt mTOR regulation, which leads to mTORC2 overactivation [34]. Therefore, a higher level of pAkt^Ser473^ in U343 MG cells sensitive to VVGMCSF- Lact may be indicative of a more efficient virus entry into cells, which is mediated by PI3K and PAK1. However, it should be noted that Akt phosphorylation at threonine 308 is required for its complete activation. The interaction between phosphatidylserine residues located on the viral membrane and the G protein-coupled receptors of the host cell gives rise to p85–p110 heterodimers (PI3K). Then, PI3K converts phosphatidylinositol 4,5-bisphosphate (PIP2) to phosphatidylinositol 3,4,5-trisphosphate (PIP3). After binding of the Akt plextrin homology domain to PIP3, Akt is phosphorylated at Thr308 by PDK1 kinase [35, 36]. The level of phosphorylated Akt was higher in U87 MG cells, which are more resistant to VV-GMCSF-Lact, both at the control point and at different incubation times with the virus, which may indicate that there is a higher level of fully activated Akt in these cells. Akt phosphorylated at the two sites activates the mTORC1 complex both indirectly, via TSC2 inactivation, and directly, via PRAS40 phosphorylation [37]. These processes lead to enhancement of protein synthesis and inhibition of the apoptosis system. Akt regulates apoptotic processes by inhibiting caspases-9 and -3 [38]. In this case, VV-GMCSF-Lact-expressed lactaptin induces apoptotic cell death via the mitochondrial pathway. Incubation of MCF-7 human breast adenocarcinoma cells with the recombinant lactaptin analog RL2 was shown to increase the level of active caspase-9 in the cells [39]. Thus, the resistance of U87 MG glioblastoma cells to VV-GMCSF-Lact can be related to the activity of the Akt kinase that inhibits lactaptin-induced apoptosis.

**p85α, p110α, pAkt^Ser473^, pAkt^Thr308^, and pPAK1^Ser199/204^ protein levels in BR1.20 and BR3.20 cells before and after exposure to the virus**

**Fig. 5:**
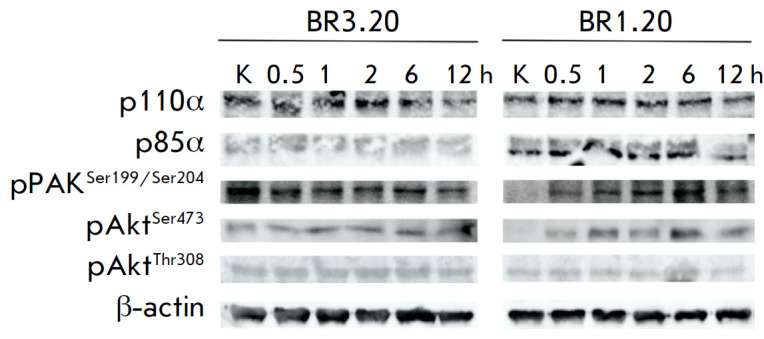
Analysis of the p85α, p110α, pAKT^Ser473^, pAKT^Thr308^, and pPAK1Ser^199/204^ proteins in the cells of patient-derived cultures BR1.20 and BR3.20. Western blot. Lanes: K – lysates of control cells; 0.5, 1, 2, 6, and 12 h – lysates of cells incubated with VV-GMCSF-Lact for different periods of time

**Fig. 6:**
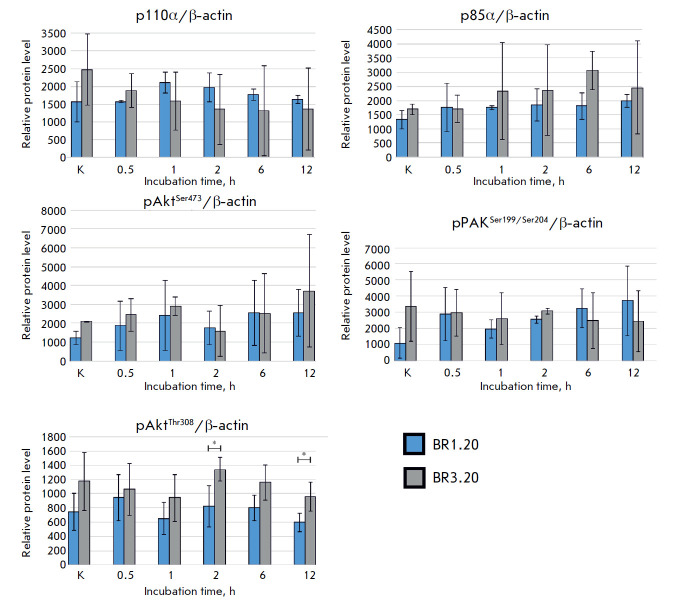
The relative levels of the p85α, p110α, pAKT^Ser473^, pAKT^Thr308^, and pPAK1^Ser199/204^ proteins in the cells of patient- derived cultures BR1.20 and BR3.20 before and after incubation with VV-GMCSF-Lact. (* *p* < 0.05)

The levels of the p85α and p110α proteins in BR1.20 and BR3.20 cells varied with virus incubation time (*Fig. 5, Fig. 6*). In BR1.20 cells, the p85α level slightly increased at 12 h of incubation. In BR3.20 cells, it increased at 6 h of incubation and then decreased after 12 h of incubation. The p110α level in BR1.20 cells remained unchanged, on average, for 12-h incubation. In BR3.20 cells, it decreased at 12 h. Meanwhile, the p110α level in BR3.20 cells, which are more resistant to VV-GMCSF-Lact, was higher at the control point (cells not exposed to the virus). The PAK1 level in more sensitive BR1.20 cells increased at 0.5 h of incubation with the virus, then decreased after 1 h, and increased again after 12 h. In contrast, the PAK1 level in BR3.20 cells decreased by 1 h of incubation with VV-GMCSF-Lact, increased by 2 h, and decreased again after 12 h. The amount of pAKT^Ser473^ in the cells of both cultures increased after 1 h of incubation, decreased at 2 h, and increased again after 12 h. On the contrary, the pAktThr308 level in cells of both cultures decreased at 1 h of incubation, increased after 2 h, and decreased after 12 h (*Fig. 3, Fig. 4*). However, by 2 and 12 h of incubation with VV-GMCSF-Lact, the pAktThr308 level was significantly higher in BR3.20 cells, which are more resistant to the virus. Thus, the levels of p85α, p110α (PI3K regulatory and catalytic subunits, respectively), pAkt^Ser473^, and pPAK1^Ser199/204^ change with incubation time, but do not vary considerably in BR1.20 and BR3.20 cells. However, it is important to note that the pAktThr308 level in BR3.20 cells, which are more resistant to the virus, was higher and significantly differed from that in BR1.20 cells by 2 h and 12 h of incubation with the virus.

Therefore, the molecular mechanisms regulating PI3K and PAK1 activation and subsequent virus entry into tumor cells may vary significantly in different cellular models of glioblastoma: in immortalized and patient-derived cell cultures. The processes controlling the efficiency of vaccinia virus entry into the cell can also involve other molecular events. For example, glioblastoma cells often contain deletions or mutations in the gene of the tumor suppressor PTEN, which, in turn, inhibits Akt activation [40, 41]. Glioblastoma cells are also characterized by a high expression of PDK1 that is able to activate PAK1 and is involved in cytoskeleton reorganization processes [42, 43, 44].

In addition, the efficiency of VV-GMCSF-Lact entry BR3.20 BR1.20 into glioblastoma cells does not mean that the virus would exhibit pronounced oncotoxic activity, because the virus needs to successfully replicate in the cell to achieve the cytotoxic effect. VV-GMCSF-Lact contains deletions in the viral thymidine kinase and growth factor genes, which prevents it from replicating in healthy, normally dividing cells. However, tumor cells may also differ in levels of growth factors and other proteins required for viral DNA replication and subsequent assembly of new viral particles, which determines the antitumor efficacy of VV-GMCSF-Lact towards different tumors.

Given these findings, we may suggest that human glioblastoma cells resistant to VV-GMCSF-Lact are characterized by an increased level of activated Akt kinase inhibiting the mitochondrial pathway of apoptosis, which probably reduces the cytotoxic effect of the recombinant virus expressing the transgene lactaptin that is an inductor of the mitochondrial apoptotic pathway.

## CONCLUSIONS

Our results suggest that human glioblastoma cells sensitive to the oncolytic virus VV-GMCSF-Lact are characterized by a low level of disturbance in the programmed cell death cascade. The mechanisms of glioblastoma resistance to standard therapy are being intensively studied. However, the issue of its resistance to oncolytic viruses still remains open [6, 7]. The mechanisms of action of the vaccinia virus, which was used to generate the recombinant strain VV-GMCSFLact, are well studied. The processes occurring in the host cell upon pathogen entry were also investigated. Tumor cells, and CSCs in particular, are characterized by disturbances in many signaling pathways, regulation of the cell cycle, and cascades of programmed cell death. A detailed study of the mechanisms contributing to the efficacy of VV-GMCSF-Lact-based therapy will help identify potential markers of tumors that are sensitive to the virus and possible targets for combined therapy with VV-GMCSF-Lact.

## Abbreviations

CSC: cancer stem cell
Akt: serine/threonine kinase 1
GM-CSF: granulocyte-macrophage colony-stimulating factor
PI3K: phosphoinositide 3-kinase
PAK1: p21-activated kinase
MEM: Minimum Essential Medium
FBS: fetal bovine serum
CD15 (Lewis X): 3-fucosyl-N-acetyllactosamine
CD171: neural cell adhesion molecule L1 (L1CAM)
PE: phycoerythrin
IC50: virus concentration causing 50% cell death
CD133: prominin-1
CD44: integral cellular glycoprotein
VACV: vaccinia virus
